# Erratum to: Physics and applications of quantum dot lasers for silicon photonics

**DOI:** 10.1515/nanoph-2022-0122

**Published:** 2022-03-18

**Authors:** Frédéric Grillot, Justin C. Norman, Jianan Duan, Zeyu Zhang, Bozhang Dong, Heming Huang, Weng W. Chow, John E. Bowers

**Affiliations:** LTCI, Télécom Paris, Institut Polytechnique de Paris, 19 Place Marguerite Perey, Palaiseau 91120, France; Center for High Technology Materials, University of New Mexico, Albuquerque 87106, NM, USA; Materials and Electrical and Computer Engineering Departments, University of California, Santa Barbara 93106, California, USA; Sandia National Laboratories, Albuquerque 87185, NM, USA

After the publication of this article, the authors found out a simple error and hereby correct them as delineated below.

(1) The former tells us that depending on the value of the coefficient *X*, the optical feedback induced frequency shift can exhibit two different behaviors: in the low feedback regime (*X* < 1), Eq. (19) has only one solution whereas for *X* < 1, Eq. (19) has more than one solution due to the increase of external cavity modes (ECM), in a number increasing with *X* hence increasing the modal competition and laser instabilities.

This description is incorrect and thereby should be replaced by a correct description as follows

The former tells us that depending on the value of the coefficient *X*, the optical feedback induced frequency shift can exhibit two different behaviors: in the low feedback regime (*X* < 1), Eq. (19) has only one solution whereas for *X* > 1, Eq. (19) has more than one solution due to the increase of external cavity modes (ECM), in a number increasing with *X* hence increasing the modal competition and laser instabilities.

